# What do general adult psychiatry patients think we should call borderline personality disorder? A cross-sectional study to find the most acceptable diagnostic term

**DOI:** 10.1192/bjb.2025.10128

**Published:** 2026-02

**Authors:** David Hayward, Amy Fourie, Donald MacIntyre, Douglas Steele

**Affiliations:** 1 Department of General Adult Psychiatry, NHS Lothian, St John’s Hospital, Livingston, UK; 2 Department of Clinical Neurosciences, University of Edinburgh, Edinburgh, UK; 3 Department of Neuroscience, University of Dundee, Dundee, UK

**Keywords:** Borderline personality disorder, stigma, diagnostic terminology, emotional intensity disorder

## Abstract

**Aims and method:**

To determine the most acceptable term for borderline personality disorder (BPD). We conducted a cross-sectional study of patients who know what it feels like to be diagnosed with a mental disorder. The main outcome measures were the proportion of participants offended and confused by alternative terms for BPD.

**Results:**

Seventy-two people participated in the study. Being diagnosed with a condition was more offensive than being diagnosed with a disorder (*χ*^2^ = 41.18, d.f. = 1, *P* < 0.01). Fluxithymia offended the fewest participants (13%), but was the most confusing term (31%). Emotionally unstable personality disorder was the most offensive term (63%). After fluxithymia, emotional intensity disorder was the least offensive term, and not especially confusing (11%). Changing BPD to emotional intensity disorder would avoid an offensive event every 3.6 diagnostic announcements.

**Clinical implications:**

The diagnostic term BPD should be replaced with emotional intensity disorder, because this term provides a balance of clarity and inoffensiveness.

Diagnostic terms are consequential. Besides facilitating communication between professionals, diagnoses direct patients’ enquires and influence their self-understanding.^1^ Moreover, categorising someone by setting their experiences in an established classification system gives them new ways to think about themselves and their pasts,^2^ and this can be self-fulfilling (‘looping effect’).^3^

As well as influencing how patients think and feel about themselves, diagnostic terms influence how others perceive them,^4^ including, potentially, having negative effects on care providers. This is especially the case for borderline personality disorder (BPD) where the label itself has more influence on clinicians than the behaviours it is supposed to reflect. To illustrate, when 265 clinicians, including psychiatrists, psychologists, social workers, community psychiatric nurses and university mental health students, were randomly given one of three written descriptions of a patient – (a) their personal details and background, (b) these details plus a description of behaviour consistent with BPD and (c) these details plus notification of a diagnosis of BPD – and they were then asked to watch a video of the woman describing her panic disorder, and then rate her present problems and likely prognosis, the clinicians’ judgements were negatively influenced by the label BPD, but not by the description of behaviours consistent with BPD, or what was shown in the video. The provision to some clinicians of the BPD diagnostic term produced more pessimistic views about the prognosis of the panic disorder and bestowed more negative views of the patient, including how motivated she was to change (BPD label < no BPD label and control, no BPD label = control; *F*(2, 250) = 4.67; *P* < 0.01).^5^

Moreover, the term BPD is anachronistic and the only diagnostic term among several hundred in the DSM ‘whose label provides no hint, no semantic handle, as to what sort of condition it is’.^6^

This study then aimed to experimentally evaluate what the preferred term for BPD should be. We were informed by a neurology study that asked out-patients which term they would prefer for the conditions that were previously called psychogenic, hysterical and medically unexplained. That study helped establish ‘functional neurological disorder’ as the preferred term, a change that improved the management of these conditions.^7^

## Method

### Patient and public involvement

The study received research ethics committee approval (London Bridge Research Ethics Committee; approval number 24/PR/0221) and was developed with the assistance of the NHS Research Scotland, Mental Health Network, Peer Researcher and Patient and Public Involvement Co-Lead, and the St John’s Hospital, Ward 17 (General Adult Psychiatry) Community Group. This included proposing and approving phrases for the questionnaire that describe how diagnostic terms can make recipients feel, and reviewing drafts of the manuscript.

### Short list of alternative diagnostic terms

**Fig. 1:**
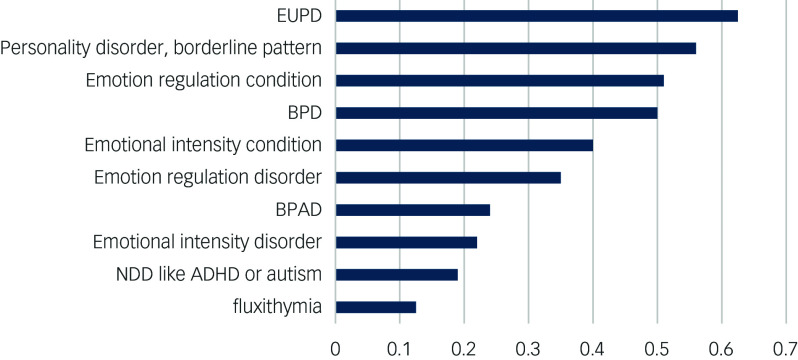
Proportion of responses conveying offence. ADHD, attention-deficit hyperactivity disorder; BPAD, bipolar affective disorder; BPD, borderline personality disorder; EUPD, emotionally unstable personality disorder; NDD, neurodevelopmental disorder.

**Fig. 2:**
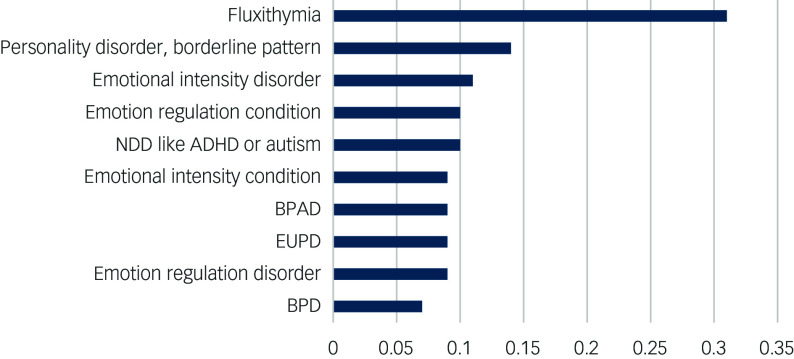
Proportion of ‘don’t understand’ responses. ADHD, attention-deficit hyperactivity disorder; BPAD, bipolar affective disorder; BPD, borderline personality disorder; EUPD, emotionally unstable personality disorder; NDD, neurodevelopmental disorder.

A short list of alternative diagnostic terms (Figs 1 and 2) was determined as follows:

The American National Institute of Mental Health supports finding a new name for BPD. Correspondingly, some institutions offering treatment programmes for people with BPD condone ‘emotional intensity disorder’ because they have determined it is popular among patients, or ‘emotional regulation disorder’ because it is popular among clinicians.^8,9^

‘Condition’ and ‘disorder’ were added as alternative postfixes because they have different implications. For example, several self-advocacy and identity movements associated with autism spectrum disorders (ASDs) have called for ASD to be considered a condition rather than a disorder.^10^

Fluxithymia was coined by authors who criticise the association of BPD with other personality disorders and encourage its classification with mood disorders.^11^ An alternative aetiological theory prioritises an empathy difference,^12^ which would align BPD with neurodevelopmental disorders (NDDs). However, diagnostic terms should avoid inferring an aetiology where the aetiology is unconfirmed (see hysterical and conversion disorders^13^), but fluxithymia and NDD were included for comparison.

In a vignette study investigating motivations for social rejection caused by mental illness diagnoses, bipolar affective disorder (BPAD) was generally perceived of positively, and it did not differ on either perceived negativity or severity with melanoma.^14^ BPAD was included then as a mental disorder diagnosis with relatively few negative connotations.

Finally, the terms used in standard clinical practice were included: BPD as per the DSM, and emotionally unstable personality disorder (EUPD) and personality disorder, borderline pattern, as per the ICD-10 and ICD-11.

### Data collection

To collect the opinions of a ‘real-world’ sample of people who know what it feels like to be given a mental disorder diagnosis that is not substantiated by investigations or test results, a cross-sectional sample (as per STROBE guidelines,^15^ *N* = 72) of general adult psychiatry patients, including in-patients (*n* = 32, eight declined), clinical psychology out-patients (*n* = 19, two declined) and general adult psychiatry out-patients (*n* = 21, two declined) were invited to participate. A consultant psychiatrist, resident doctor and five medical students used a questionnaire (Appendix) to determine whether the names in the short list had offensive connotations or were not understood. The symptom list in the questionnaire was based on the DSM-IV-TR BPD diagnostic criteria.^16^ Responses were coded as either ‘yes’, ‘no’ or ‘don’t understand’ for each diagnosis and connotation.

The proportion of participants who endorsed one or more of the offensive responses (‘you are badly behaved’ and ‘you should pull yourself together’) was determined, as was the proportion of participants who endorsed the ‘don’t understand’ response to one or more of the responses.

## Results

Primary data is shown in Table 1.

**Table 1:** Diagnostic terms and responses

		Responses											
		You are badly behaved – agree	You are badly behaved – disagree	You are badly behaved – don’t understand	You legitimately have a mental health problem – agree	You legitimately have a mental health problem – disagree	You legitimately have a mental health problem – don’t understand	You should pull yourself together – agree	You should pull yourself together – disagree	You should pull yourself together – don’t understand	You deserve help and treatment – agree	You deserve help and treatment – disagree	You deserve help and treatment – don’t understand
Diagnostic terms	Emotion regulation disorder	21	46	5	46	17	9	29	36	7	51	16	5
	Emotional intensity disorder	14	48	10	44	12	6	18	43	11	59	8	5
	Fluxithymia	9	37	26	34	19	19	9	37	26	45	9	18
	BPD	40	27	5	42	27	3	32	31	9	57	11	4
	EUPD	48	19	6	44	23	5	42	6	10	51	17	4
	Personality disorder, borderline pattern	45	19	7	38	26	8	35	24	13	49	15	8
	BPAD	19	41	12	62	8	2	15	47	10	48	3	1
	NDD	19	44	9	61	7	4	9	52	11	64	3	5
	Emotion regulation condition	27	38	6	41	24	6	46	26	10	54	10	8
	Emotional intensity condition	24	41	7	43	25	4	33	31	8	56	9	7

BPD, borderline personality disorder; EUPD, emotionally unstable personality disorder; BPAD, bipolar affective disorder; NDD, neurodevelopmental disorder.

In this diverse sample of people who know what it feels like to be given a subjective mental disorder diagnosis, 19% would be offended if they presented with BPD symptoms and were diagnosed with an NDD; 24% would be offended if they presented with BPD symptoms and were diagnosed with BPAD. This is perhaps as acceptable as a subjective mental disorder diagnosis can be.

The proportions of offended participants are shown in Fig. 1, and the proportions of confused participants are shown in Fig. 2.

Being diagnosed with a condition was considered significantly more offensive than being diagnosed with a disorder (*χ* ^2^ = 41.18, d.f. = 1, *P* < 0.01).

Fluxithymia offended the fewest participants (13%), but was the most confusing term (31%). It was significantly more confusing than even the next most confusing term, which was personality disorder, borderline pattern (*χ* ^2^ = 28.70, d.f. = 1, *P* < 0.01).

The established terms were among the most offensive. EUPD offended 63% of participants; personality disorder, borderline pattern offended 56% of participants; and BPD offended 50% of participants.

According to our results, if the diagnostic term was changed from BPD to emotional intensity disorder, an offensive event would be avoided every 3.6 diagnostic pronouncements (absolute risk reduction = (control event rate 50%) − (experimental event rate 22%), and number needed to treat = 1/absolute risk reduction). Similarly, if personality disorder, borderline pattern is the diagnostic term used and it was replaced with emotional intensity disorder, a patient would avoid being offended by their diagnosis every 2.9 diagnostic pronouncements (number needed to treat 2.9), and replacing EUPD with emotional intensity disorder would avoid a patient being offended by their diagnosis every 2.5 diagnostic pronouncements (number needed to treat 2.5).

### Limitations

The sample size was moderate, only included people engaged in treatment and was drawn from a single site. However, the results were statistically significant, various patients participated and the site was a district general hospital that serves a socioeconomically diverse population. The responses to the questionnaire were forced choice, which made for actionable results, but did not allow neutral or indifferent answers. The diagnoses the participants had were not recorded, and we did not compare the results of the different subpopulations (psychiatry in-patients versus psychiatry out-patients versus psychology out-patients). Different proportions of these subpopulations would have had BPD.

Dyslymbia has also been proposed as an alternative diagnostic term,^17^ but we did not include it because we thought it would have the same disadvantages as fluxithymia.

## Discussion

Usually, clinical terminology is decided without considering the sensibilities of the people it will apply to. For example, the diagnoses in the major classification systems were formulated by predominantly academic committees and then tested in field studies for validity and reliability, but not acceptability. There are examples too of clinical terms being changed mindful of the impact on affected people, but without actually asking them. For example, it was assumed that patients would prefer to be called ‘service users’ but when opinions were surveyed, it turned out they did not.^18^ There have only been a few worthy attempts to refine diagnostic terms in order to lessen their impact on patients, with the quoted neurology study being an example.^7^

Unexpectedly, the term disorder was preferred to the term condition. Several participants explained that this was because condition sounded ‘wishy-washy’ or too mild a term.

The established diagnostic labels of BPD, EUPD and personality disorder, borderline pattern were among the most offensive (and of these, the most recent – personality disorder, borderline pattern – was the most confusing). Participants explained that this was because these labels appear to besmirch someone’s whole persona. However, contrary to expectations, the prefix ‘borderline’ was not especially confusing because, conversations with participants revealed, it was misinterpreted as simply conveying a milder type of personality disorder.

Although a new term for an established disorder might eventually take on the stigma that came with the term it replaced, adopting a new term provides an opportunity to reconceive the underlying mechanisms.

We contend, of the alternative diagnostic terms for BPD, emotional intensity disorder should be adopted because it provides a balance of clarity and inoffensiveness and does not infer an aetiology or classification, and so avoids implicating a particular treatment.

## Data Availability

The data that support the findings of this study are available from the corresponding author upon resonable request.

## About the authors

**David Hayward** is a consultant psychiatrist at the Department of General Adult Psychiatry, NHS Lothian, St John’s Hospital, Livingston, UK. **Amy Fourie** is a resident doctor at the Department of General Adult Psychiatry, NHS Lothian, St John’s Hospital, Livingston, UK. **Donald MacIntyre** is a consultant psychiatrist in General Adult Psychiatry, NHS Lothian, and an honorary reader at the Department of Clinical Neurosciences, University of Edinburgh, Edinburgh, UK. **Douglas Steele** is a professor of neuroimaging at the University of Dundee, Dundee, UK, and an honorary consultant psychiatrist, NHS Tayside, Dundee, UK.

## Appendix

Please imagine you were referred to a mental health professional because of issues like – ‘lots of ups and downs in relationships, impulsivity, very changeable moods, angriness that made you lose control, sometimes hurting yourself or feeling suicidal, changing your mind depending on who you are with, feeling bored or empty, & feeling frantic when you think someone you value might leave you’

– and you were given one of the diagnoses in the blue column, would that make you think any of the things in the orange row?
